# The alkynyl-containing compounds from mushrooms and their biological activities

**DOI:** 10.1007/s13659-023-00416-w

**Published:** 2023-11-10

**Authors:** Ji-shuang Qi, Yingce Duan, Zhao-chen Li, Jin-ming Gao, Jianzhao Qi, Chengwei Liu

**Affiliations:** 1 Key Laboratory for Enzyme and Enzyme-Like Material Engineering of Heilongjiang, College of Life Science, Northeast Forestry University, Harbin, 150040 China; 2 Shaanxi Key Laboratory of Natural Products & Chemical Biology, College of Chemistry & Pharmacy, Northwest A&F University, Yangling, 712100 China

**Keywords:** Mushroom, Alkynyl compounds, Bioactivity, Chemical structures

## Abstract

Mushrooms have been utilized by humans for thousands of years due to their medicinal and nutritional properties. They are a crucial natural source of bioactive secondary metabolites, and recent advancements have led to the isolation of several alkynyl-containing compounds with potential medicinal uses. Despite their relatively low abundance, naturally occurring alkynyl compounds have attracted considerable attention due to their high reactivity. Bioactivity studies have shown that alkynyl compounds exhibit significant biological and pharmacological activities, including antitumor, antibacterial, antifungal, insecticidal, phototoxic, HIV-inhibitory, and immunosuppressive properties. This review systematically compiles 213 alkynyl-containing bioactive compounds isolated from mushrooms since 1947 and summarizes their diverse biological activities, focusing mainly on cytotoxicity and anticancer effects. This review serves as a detailed and comprehensive reference for the chemical structures and bioactivity of alkynyl-containing secondary metabolites from mushrooms. Moreover, it provides theoretical support for the development of chemical constituents containing alkynyl compounds in mushrooms based on academic research and theory.

**Graphical Abstract:**

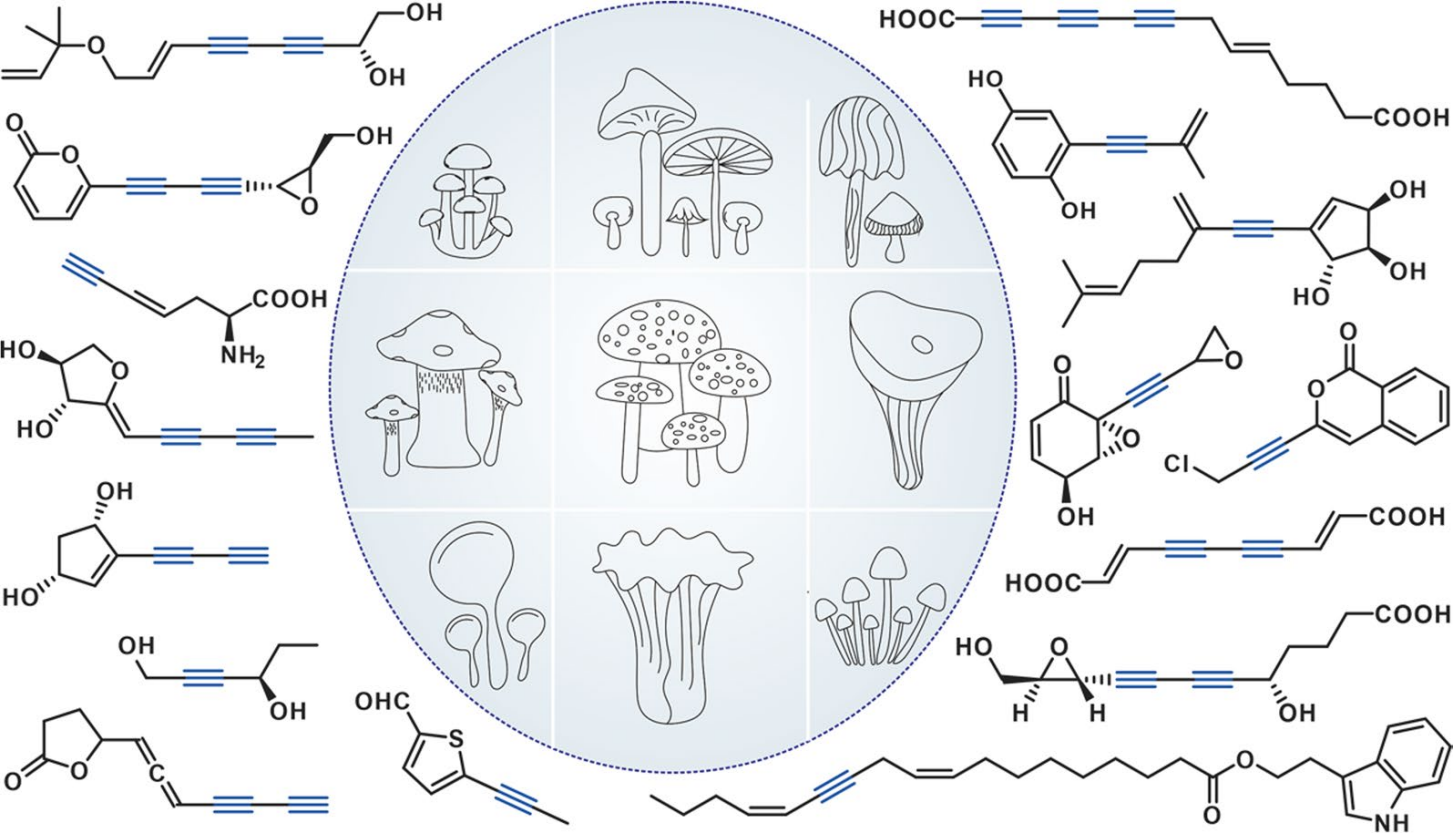

## Introduction

### Mushroom

Mushrooms are classified as macrofungi*,* exhibiting prominent fruiting bodies that are visible to the naked eye and can be handpicked [1]. The fruiting bodies of basidiomycetes and ascomycetes are called “mushrooms”. It produces spores, which germinate and produce mycelium. The mycelium ultimately produces the primordium, which grows into a new complete mushroom and its life cycle continues. Apart from their historical significance as a food source, mushrooms hold considerable importance in traditional medicine due to their healing properties [2]. For millennia, medicines and natural products have been closely linked through the use of traditional medicines and natural poisons. Mushrooms, with their extensive history in traditional Eastern medicine, often serve as valuable supplements in medicinal preparations. Medicinal mushroom preparations offer favorable health benefits, without any apparent adverse side effects, allowing for regular and moderate usage without causing harm. Mushrooms constitute a vast and untapped reservoir of new medicinal products. Of particular significance to modern medicine is its abundant supply of compounds that serve as regulators of tumor cell growth. Earth hosts an estimated 140,000 mushroom species, with only 10% identified [3].

Mushrooms are composed of water (85–95%), carbohydrates (35–70%), proteins (15–34.7%), fats (< 10%), minerals (6–10.9%), nucleic acids (3–8%), and vitamins [4]. Due to their carbohydrate, fiber, protein, essential amino acid, unsaturated fatty acid, mineral, and vitamin content, as well as their low-calorie count, mushrooms are recognized as a nutritious and health-promoting food [4]. In recent years, numerous secondary metabolites with medicinal potential have been isolated from mushrooms. Pharmacological studies have demonstrated that mushrooms and their metabolites exhibit antibacterial, antiviral, anti-inflammatory, anti-oxidant, antitumor, and immune-enhancing activities. Throughout history, mushrooms have been integrated into various aspects of human life, serving as food and medicine. Grounded in the concept of the homology of food and medicine, the distinction between edible and medicinal uses becomes less absolute. Numerous common edible mushrooms, such as shiitake, exhibit therapeutic effects, leading to their adoption in clinical applications. Mushrooms possess not only a delightful taste but also a healthy chemical composition, characterized by low fat, high protein content, and abundant vitamins. Moreover, mushrooms rank as low glycemic index foods with deficient sodium levels and high phosphorus and potassium content, rendering them widely applicable in dietary therapy, health care, and medical treatment [5].

### Alkynyl compound

In nature, numerous unsaturated compounds consist of molecules containing one, two, or more triple bonds. These naturally occurring compounds are commonly referred to as acetylenes, which should not be confused with the term polyacetylene, as it does not pertain to polymers. Their production encompasses metabolites and precursors that contain only three bonds. Thus, “acetylene” refers to compounds (cyclic or linear) containing an alkynyl portion, while “polyacetylene” pertains to compounds with more than one triple bond [6]. The acetylene (ethynyl) group is acknowledged as a distinctive structural element that targets a diverse array of therapeutic proteins, encompassing monoamine oxidase, tyrosine kinases, β-site amyloid precursor protein cleaving enzyme, steroid receptors, metabotropic glutamate receptor 5, free fatty acid receptor 1, and HIV-1 reverse transcriptase [7]. Metabolites containing alkynyl groups have been isolated and characterized from various sources, including plants, fungi, and other organisms [8]. Naturally occurring compounds containing acetylene-based units are particularly interesting due to their significant biological activities, including antitumor, antibacterial, and antifungal properties. Mushrooms contain a significant variety of compounds with alkynyl groups. Currently, 213 compounds have been isolated and extracted from basidiomycota and ascomycetes, demonstrating antibacterial and antifungal activities (Table 1). This article presents a comprehensive summary of alkynyl compounds found in mushrooms since 1947. The compounds are categorized based on their carbon (C) content, including those with 10 or fewer C atoms, those featuring amino or amino acids at the end, those with *O*-isopentenyl at the end, those with epoxy ring, those with more than 10 C atoms, those with lactone ring, those with six-membered ring structure, those with indole ring, those with sulfur (S), oxygen(O) structure, both lactone ring and epoxy ring, and those with a five-ring structure. This review is the first systematic compilation of the structural characteristics and biological activities of alkynyl-containing compounds in mushrooms, providing valuable references for drug activity and biosynthesis.

**Table 1:** Name,bioactives and source

Number	Name	Bioactivity	Source	Reference
**1**	1-Hydroxy-2-nonyn-4-one	Antifungi	*I. benzoinum*	[9]
**2**	Acetylenic diol		*C. catinus*	[10]
**3**			*F. annosus*	[11]
**4**	Hepta-4,6-diyn-3-ol	Antibacterial	*G. spectabilis*	[12]
**5**	7-Chloro-hepta-4,6-diyn-3-ol	Antibacterial	*G. spectabilis*	[12]
**6**	Scobinynediol-II		*P. scobinacea*	[14]
**7**	Scobinynediol-II		*P. scobinacea*	[14]
**8**	Scobinynediol-I		*P. scobinacea*	[14]
**9**	Octa-2,4,6-triynoic acid		*P. scobinacea*	[14]
**10**	Triynol		*P. merdaria*, *K. mutabilis*, *R. vesca*, *R. flava*,	[13]
**11**	Triyne acid		*P. merdaria*, *K. mutabilis*, *R. vesca*, *R. flava*,	[13]
**12**	Triyne alcohol		*P. merdaria*, *K. mutabilis*, *R. vesca*, *R.flava*	[13]
**13**	Polyacetylenic acid		*C. virgineus*	[15]
**14**			*P. sinuosa*, *Coprinus* species	[16, 17]
**15**			*P. sinuosa*	[16]
**16**		Nematicidal activity	*P. cocos*	[18]
**17**			*P. sinuosa*	[16]
**18**	Lentinamycin	Antibacterial	*L. edodes*	[19–23]
**19**	Pyranone derivative B	Cytotoxicity	*J. nitida*	[24]
**20**			*F. pallida*	[25]
**21**			*F. pallida*	[25]
**22**			*S. lacrymans*	[13]
**23**	(*Z*)-non-7-en-5-yn-1,2,4-triol		*T. pardinum*	[26]
**24**	(*Z*)-non-7-en-5-yn-1,4-diol		*T. pardinum*	[26]
**25**	(*Z*)-1,2-dihydroxynon-7-en-5-yn-4-one		*T. pardinum*	[26]
**26**	(*Z*)-1-hydroxynon-7-en-5-yn-4-one		*T. pardinum*	[26]
**27**	Allenediyne (−)-marasin	Antibacterial, antimycobacterial	*M. ramealis*	[27, 28]
**28**	Scorodonin	Antibacterial, antifungi, nucleotide conjugation inhibitory activity	*M. scorodonius*	[29]
**29**		Antifungal	*L. edodes*	[21, 22]
**30**			*L. edodes*	[21]
**31**			*L. edodes*	[21]
**32**		Antifungal	*L. edodes*	[21, 22]
**33**	(2*E*)-decane-4,6,8-trienoic acid	Anti-oxidant	*L. decastes*	[30]
**34**	(2*E*)-decene-4,6,8-triyn-1-ol	Anti-oxidant	*L. decastes*, *H. marmoreus, P. sinuosa*, *L. lepideus*, *L. giganteus*, *L. dacastes, P. resinosa*	[16, 30–32]
**35**	4,6,8-Decatriyn-1-ol		*H. marmoreus*, *P. scobinacea*	[14, 31]
**36**			*H. marmoreus*	[33]
**37**	(3*S*,8*S*)-(−)-4,6-decadiyne-l,3,8-triol		*G. spectabilis*	[34]
**38**			*F. pallida*	[25]
**39**			*F. pallida*	[25]
**40**			*C. virgineus*	[15]
**41**			*C. virgineus*	[15]
**42**			*C. virgineus*	[15]
**43**			*P. sinuosa*	[16]
**44**			*P. sinuosa*	[16]
**45**			*P. sinuosa*	[16]
**46**			*P. sinuosa*	[16]
**47**			*L. lepideus*, *L. giganteus*, *L. dacastes, P. resinosa*	[32]
**48**	Masutakic acid A		*L. sulphureus*	[35]
**49**			*S. lacrymans*	[13]
**50**			*S. lacrymans*	[13]
**51**			*S. lacrymans*	[13]
**52**		Antibacterial	*C. catinus*	[10]
**53**		Antibacterial	*C. catinus*	[10]
**54**	Triynol		*P. scobinacea*	[14]
**55**	Chondrosterin G		*Chondrostereum* species	[36]
**56**	Chondrosterin H		*Chondrostereum* species	[36]
**57**	Diatretyne amide	Antibacterial	*C. diatreta*	[37]
**58**	Diatretyne nitrile	Antibacterial,	*C. diatreta, C. virgineus*	[15, 37]
**59**	(2*S*)-2-amino-4-pentynoic acid		*A. pseudoporphyria*	[38]
**60**	(2*S*)-2-aminohept-4-en-6-ynoic acid		*A. pseudoporphyria*	[38]
**61**	(2*R*)-2-amino-6-hydroxy-4-hexynoic acid		*A. miculifera*	[39]
**62**	2-amino-4-pentynoic acid	Inhibit fatty acid oxidation	*A. abrupta*	[40]
**63**	(2*S*,3*R*)-2-amino-3-hydroxypent-4-ynoic acid	Toxic to chickens	*S. rolfsii*	[41]
**64**	(2*S*)-2-amino-4-hexynoic acid		*T. rutilans*	[42]
**65**	(2*S*)-2-amino-3-hydroxy-4-hexynoic acid		*T. rutilans*	[43]
**66**	γ-l-Glutamyl-(2*S*)-2-amino-4-hexynoic acid		*T. rutilans*	[44]
**67**	γ-Glutamyl-(2*S*,3*S*)-2-amino-3-hydroxy-4-hexynoic acid		*T. rutilans*	[44]
**68**	γ-Glutamyl-l-2-aminohex-4-ynoic acid		*T. rutilans*	[44]
**69**	γ-l-Glutamyl-l-erythro-2-amino-3-hydroxyhex-4-ynoic acid		*T. rutilans*	[44]
**70**	(2*S*)-2-amino-5-hexynoic acid	Antibacterial	*C. claricolor*	[45]
**71**	Agrocybin	Active against TryR, cytotoxicity, toxic to white rats, phytotoxic, antibacterial, antifungal	*A. perfecta*, *A. dura, C. formosus*	[46–48]
**72**	Agrocybyne A	Inhibited lettuce	*A. praecox*	[49]
**73**	Agrocybyne B	Inhibited lettuce	*A. praecox*	[49]
**74**	Agrocybyne C	Inhibited lettuce	*A. praecox*	[49]
**75**	Agrocybyne D	Inhibited lettuce	*A. praecox*	[49]
**76**	Agrocybyne E		*A. praecox*	[49]
**77**	10-Hydroxyundeca-2,4,6,8-tetraynamide	Antibacterial, antifungal	*M. viridimarginata*	[50]
**78**	(2*R*)-amino-4S-hydroxy-5-hexynoic acid	Toxic to mice	*T. venenata*	[51]
**79**	(2*R*)-amino-5-hexynoic acid	Toxic to mice	*T. venenata*	[51]
**80**			*P. sinuosa*	[16]
**81**	Isoprenyl ether		*F. bisphaerigera*	[52]
**82**	(*E*)-10-(1,1-dimethyl-2-propenyloxy)-2-decene-4,6,8-triyn-1-ol		*H. marmoreus*	[31]
**83**	10-(1,1-dimethyl-2-propenyloxy)deca-4,6,8-triyn-1-ol		*H. marmoreus*	[31]
**84**	Nemotinic acid	Antibacterial, antimycobacterial	Basidiomycete B-841	[28, 53–57]
**85**			*L. lepideus, L. giganteus, L. dacastes, P. resinosa*	[32]
**86**			*L. lepideus, L. giganteus, L. dacastes, P. resinosa*	[32]
**87**			*C. berkeleyanus*	[20]
**88**			*P. sinuosa*	[16]
**89**	Tetrayne tetraol		*F. hepatica*	[25]
**90**	Feldin		*F. hepatica*	[58]
**91**	4-Dodecene-6,8-diyne-1,3,10-triol		*F. hepatica*	[58]
**92**	Falcarinol		*F. hepatica*	[58]
**93**	Falcarindiol		*F. hepatica*	[58]
**94**	Oenanthetol		*F. hepatica*	[58]
**95**	Triynene diol		*F. pallida*	[25]
**96**	Phomallenic acid A	Fatty acid synthesis inhibitory	*Phoma* species	[59]
**97**	Phomallenic acid B	Fatty acid synthesis inhibitory	*Phoma* species	[59]
**98**	Mycomycin		*C. formosus*	[47]
**99**	14-Oxo-9,15-octadecadien-12-ynoic acid		*C. cibarius*	[60]
**100**	14-Oxo-9,15-octadecadien-12-ynoic acid methyl ester		*C. cibarius*	[60]
**101**	Dehydromatrine ethyl ester	Anti-oxidant	*L. decastes*	[30]
**102**	Dehydromatricaria acid		*P. scobinacea*	[14]
**103**			*P. scobinacea*	[14]
**104**			*Polyporus* species	[61]
**105**			*Polyporus* species	[61]
**106**	(8*E*,10*R*,14*Z*)-10-hydroxy-8,14-octadecadien-12-ynoic acid		*C. aureus*	[62]
**107**	Craterellyne E		*C. lutescens*	[64]
**108**			*C. lutescens*	[64]
**109**	Craterellyne F		*C. lutescens*	[64]
**110**	Craterellyne J		*C. lutescens*	[63]
**111**	Craterellyne M		*C. lutescens*	[63]
**112**	Craterellyne P		*C. lutescens*	[63]
**113**	Phomallenic acid C		*Phoma* species	[59]
**114**	(10*E*,14*Z*)-9-hydroperoxy-10,14-octadecadien-12-ynoic acid		*C. lutescens*	[60]
**115**	(10*E*,14*Z*)-9-hydroxy-10,14-octadecadien-12-ynoic acid		*C. cibarius*	[60, 65]
**116**	(10*E*,14*Z*)-9-oxo-10,14-octadecadien-12-ynoic acid		*C. cibarius*	[60, 65]
**117**	14,17,18-Trihydroxy-9,15-octadecadien-12-ynoic acid		*C. cibarius*	[60]
**118**	9-Hydroxy-14-oxo-10,15-octadecadien-12-ynoic acid		*C. cibarius*	[60]
**119**	9-Hydroperoxy-14-oxo-10,15-octadecadien-12-ynoic acid		*C. cibarius*	[60]
**120**	9,14-Dioxo-10,15-octadecadien-12-ynoic acid		*C. cibarius*	[60]
**121**	Ximeninic acid		*C. cibarius*	[66]
**122**	Craterellyne A		*C. lutescens*	[64]
**123**	Craterellyne B		*C. lutescens*	[64]
**124**	Craterellyne C		*C. lutescens*	[64]
**125**	Craterellyne K		*C. lutescens*	[63]
**126**	Craterellyne O		*C. lutescens*	[63]
**127**	Craterellyne N		*C. lutescens*	[63]
**128**	Craterellyne L		*C. lutescens*	[62, 63]
**129**	Craterellyne Q		*C. lutescens*	[63]
**130**	14-*O*-ethyl-craterellyne O		*C. lutescens*	[63]
**131**	14,15-Dehydrocrepenynic acid methyl ester		*C. cibarius*	[60]
**132**	14,15-Dehydrocrepenynic acid ethyl ester		*C. cibarius*	[60]
**133**	Repandum	Cytotoxic, affinity for specific nucleic acids	*H. repandum*	[27, 67]
**134**	Biforminic acid	Antibacterial	*P. biformis*	
**135**	Biformin	Antibacterial	*P. biformis*	
**136**	Craterellyne G		*C. lutescens*	[63]
**137**	Craterellyne H		*C. lutescens*	[63]
**138**	Craterellyne I	Antibacterial, cytotoxic, inhibit the production of NO	*C. lutescens*	[63]
**139**	9-*epi*-craterellyne H		*C. lutescens*	[63]
**140**	(9*Z*,15*E*)-17(18)-epoxy-14-oxo-9,15-octadecadien-12-ynoic acid methyl ester		*C. cibarius*	[60]
**141**	Pyranone derivative C	Cytotoxicity	*J. nitida*	[24]
**142**	Pyranone derivative D	Cytotoxicity	*J. nitida*	[24]
**143**	Xerulin	Inhibited the suppression of cholesterol biosynthesis	*X. melanotricha, F. hepatica*	[58, 70]
**144**	Dihydroxerulin	Inhibited the suppression of cholesterol biosynthesis	*X. melanotricha, F. hepatica*	[58, 70]
**145**	Xerulinic acid	Inhibited the suppression of cholesterol biosynthesis	*X. melanotricha, F. hepatica*	[58, 70]
**146**	Pyranone derivative A	Cytotoxicity	*J. nitida*	[24]
**147**	3,4,13-Trihydroxy-tetradeca-5,7,9,11-tetraynoic acid-γ-lactone		*M.viridimarginata*	[50]
**148**	Nemotin		*C. formosus*	[47]
**149**	Aporpinone A	Cytotoxicity	*H. speciosa*, *A. caryae*	[71, 72]
**150**	4ʹ-Hydroxyaporpinone A		*A. caryae*	[71]
**151**	50-O-acetylaporpinone A		*H. speciosa*	[72]
**152**	Tricholomenyn A	Anti-mitotic	*T. acerbum*	[73]
**153**	Tricholomenyn B	Anti-mitotic	*T. acerbum*	[73]
**154**	Tricholomenyn C		*T. acerbum*, *T.ustaloides*, *T. vaccinum*, *T.albobrunneum*, *T. imbricatum*	[74]
**155**	Tricholomenyn D		*T. acerbum*	[74]
**156**	Tricholomenyn E		*T. acerbum*	[74]
**157**	3-(Hydroxymethyl)-2,5-bis(3-methylbut-3-en-1-ynyl)benzene-1,4-diol		*S. hirsutum*	[75]
**158**	2,5-Dihydroxy-3-isoprenyl-6-(3-methylbut-3-en-1-ynyl)benzaldehyde		*S. hirsutum*	[75]
**159**	Frustulosin	Phytotoxic, inhibition of callus growth	*S. hirsutum*	[75]
**160**	Sterehirsutinal		*S. hirsutum*	
**161**	Frustulosinol		*S. hirsutum*	[75]
**162**	2-Hydroxy-5-methoxy-6-(3-methylbut-3-en-1-ynyl) benzylalcohol		*S. hirsutum*	[75]
**163**	Frustulosinol		*S. hirsutum*	[76]
**164**	Sterehirsutyne C	PPL inhibitor	*S. hirsutum*	[76]
**165**	Sterehirsutyne A	PPL inhibitor	*S. hirsutum*	[76]
**166**	Sterehirsutyne B		*S. hirsutum*	[76]
**167**	Vibrayne		*S. hirsutum*	[76]
**168**	Frustulosin	Antibacterial	*S. frustulosum*	[77]
**169**	Frustulosinol	Antibacterial	*S. frustulosum*	[77]
**170**	Cinnatriacetin A	Antimicrobial	*F. hepatica*	[78]
**171**	Cinnatriacetin B	Antimicrobial	*F. hepatica*	[78]
**172**	Mycenon	Isocitrate lyase inhibitors, antibacterial, antifungi	*Mycena species*	[79]
**173**	Mycenadiol A		*M. pruinosoviscida*	[80]
**174**	Mycenadiol B		*M. pruinosoviscida*	[80]
**175**	Siccayne	Nucleoside uptake, nucleotide incorporation interference	Ascomycetes	[81]
**176**	Speciosin A		*H. speciosa*	[82]
**177**	Speciosin B	Cytotoxicity	*H. speciosa*	[82]
**178**	Speciosin C		*H. speciosa*	[82]
**179**	Speciosin D		*H. speciosa*	[72]
**180**	Speciosin E		*H. speciosa*	[72]
**181**	Speciosin F		*H. speciosa*	[72]
**182**	Speciosin G		*H. speciosa*	[82]
**183**	Speciosin L		*H. speciosa*	[72]
**184**	Speciosin M		*H. speciosa*	[82]
**185**	Speciosin N		*H. speciosa*	[82]
**186**	Speciosin O		*H. speciosa*	[82]
**187**	Speciosin P		*H. speciosa*	[72]
**188**	Antrocamphin A	Cytotoxicity, anti-inflammatory	*A. cinnamomea, T. camphoratus*	[83, 87, 88]
**189**	Benzocamphorin F	Anti-NOS	*A. cinnamomea*	[85]
**190**	Benzocamphorin H	Anti-inflammatory	*A. cinnamomea*	[84]
**191**	4,7-Dimethoxy-5-methyl-6-(3-methylbut-3-en-1-ynyl)-1,3-benzodioxole	Anti-inflammatory	*A.cinnamomea*	[86]
**192**	Antrocamphin B		*A. camphorata*	[83]
**193**	Antrodioxolanone		*A. camphorata T. camphoratus*	[83, 88]
**194**		Antibacterial	*B. myosura*, *C. formosus*	[47, 89]
**195**	Peniophorin A	Antimicrobial	*P. affinis*	[90]
**196**	Peniophorin B	Antimicrobial	*P. affinis*	[90]
**197**			*F. Pallida*	[25]
**198**	Phenyl-lactate		*F. Pallida*	[25]
**199**	Ochroleucin A		*R. ochroleuca*	[91]
**200**	Ochroleucin B		*R. ochroleuca*	[91]
**201**	Gymnopalyne A	Antimicrobial, antifungal	*Gymnopus* species	[81]
**202**	Gymnopalyne B	Antimicrobial, antifungal	*Gymnopus* species	[81]
**203**	Benzocamphorin A		*T.camphoratus*	[88]
**204**	Benzocamphorin B		*T.camphoratus*	[88]
**205**			*C.cornucopioides*	[92]
**206**	Junipal		*D.juniperina*	[93]
**207**	3,4-Dihydroxy-2-hexyl-2,4-diimino-2-tetrahydrofuran	Anti-oxidant	*L. decastes*	[30]
**208**	Nitidon	Antibacterial, antifungal cytotoxicity	*J. nitida*	[24, 94]
**209**	Aporpinone B	Antibacterial	*A. caryae*	[71]
**210**	1ʺ-Acetylaporpinone B	Antibacterial	*A. caryae*	[71]
**211**	Stereyne A		*S. hirsutum*	[95]
**212**	Sistodiolynne		*S.raduloides*	[96]
**213**	Stereyne B		*S. hirsutum*	[95]

## The classification of alkynyl compounds in mushrooms

### Linear alkynyl with C ≤ 10

Linear alkynyl with C ≤ 10 refers to compounds containing ten carbon atoms or fewer in the long chain that includes the alkynyl group. Various modifications, such as hydroxylation and carboxylation, occur at the end of linear alkynyl. A total of 56 types of these compounds have been identified.

#### Linear alkynyl with C ≤ 9

Linear alkynyl with C ≤ 9 contain nine or fewer carbon atoms in the long chain, including the alkynyl group. The termini of linear alkynyl undergo various modifications such as hydroxylation, carboxylation. A total of 32 compounds were identified.

**Fig. 1:**
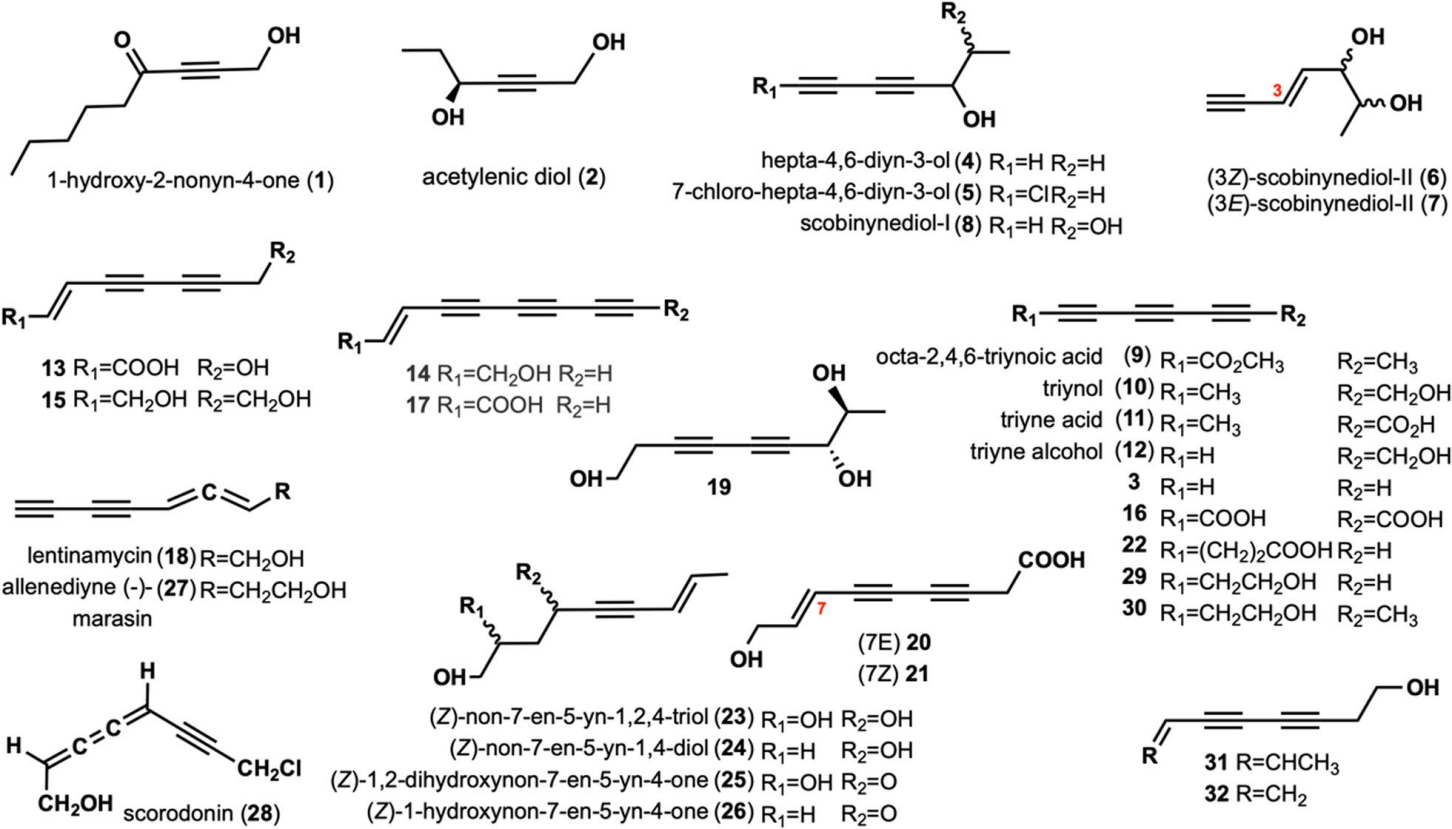
Chemical structures of linear alkynyl with C ≤ 9

1-hydroxy-2-nonyn-4-one (**1**) was isolated from *Ischnoderma benzoinum*, which shows significant inhibitory activity against yeasts and filamentous fungi at concentrations ranging from 1 to 5 μg/mL [9]. Moreover, at the same concentrations, **1** strongly inhibits nucleic acid, and protein syntheses in cells of the ascitic form of Ehrlich carcinoma [9]. Acetylenic diol (**2**) was isolated from of cultures *Clitocybe catinus* [10]. Compound **3** was isolated from *Fomes annosus* [11]. Hepta-4,6-diyn-3-ol (**4**), 7-chloro-hepta-4,6-diyn-3-ol (**5**) were isolated from *Gymnopilus spectabilis* [12], compound **5** is also one of the metabolites of *G. hybridu*s [13]. Compounds **4** and **5** exhibit antibacterial activity by agar diffusion test [12]. When tested at a concentration of 100 μL/mL per disc, compound **4** exhibits inhibition against the following bacteria including *Bacillus brevis* [12]. Scobinynediol-II (**6)**, scobinynediol-II **(7)**, scobinynediol-I **(8)** and octa-2,4,6-triynoic acid **(9)** were isolated from the culture medium *Psathyrella scobinacea* [14]. Triynol (**10**), triyne acid (**11**), and triyne alcohol (**12**) were isolated from *Psilocybe merdaria*, *Kuehneromyces mutabilis*, *Russula vesca* and *Ramaria flava* [13]. Polyacetylenic acid (**13**) was isolated from *Camarophyllus virgineus* [15]. Compounds **14** and **15** were isolated from *Poria sinuosa* [16], and Compound **14** was isolated from *Coprinus* species [17]. Compound **16** was isolated from the culture of *Poria cocos*, which exhibited significant nematicidal activity against *Meloidogyne arenaria* and *Panagrellus redivivus* [18]. Compound **17** was isolated from *P. sinuosa* [16]. Lentinamycin (**18**) was isolated from *Lentinula edodes*, demonstrating antibacterial activity against common bacterias and pathogenic bacteria in an antibacterial activity test using the paper disk method (31.3 nM/disk) [19–22]. Compound **18** also showed antimicrobial activity against a variety of filamentous fungi such as *Aspergillu*s and yeasts, as well as anti-gram-positive bacterial activity with the minimum inhibitory concentration (MIC) value of 0.085–0.42 μM [19, 23]. Pyranone derivative B (**19**) was isolated from *Junghuhnia nitida*. It displayed cytotoxicity against five human cancer cell lines, including human myeloid leukemia HL-60, hepatocellular carcinoma SMMC-7721, lung cancer A-549, breast cancer MCF-7, and colon cancer SW480, with IC_50_ > 40 μM [24]. Compounds **20** and **21** were isolated from *Fistulina pallida* [25]. Compound **22** was isolated from *Serpula lacrymans* [13]. (*Z*)-non-7-en-5-yn-1,2,4-triol (**23**), (*Z*)-non-7-en-5-yn-1,4-diol (**24**), (*Z*)-1,2-dihydroxynon-7-en-5-yn-4-one (**25**), (*Z*)-1-hydroxynon-7-en-5-yn-4-one (**26**) were isolated from *Tricholoma pardinum* [26]. The allenediyne (-)-marasin (**27**) was extracted as an active antibiotic component against *S. aureus* from a culture of *Marasmius ramealis* in 1959, and was the first naturally occurring olefin to be isolated [27]. Compound **27** was also found to exhibit antibacterial and antimycobacterial activities [28]. Scorodonin (**28**) is a bioactive metabolite isolated from *Marasmius scorodonius*, which exhibits inhibitory effects on the growth of bacteria, yeast, and filamentous fungi [29]. Compound **28** also showed significant inhibition of nucleic acid [29]. Compounds (**29**)–(**32**) were isolated from *L. edodes* [21]. Among them, compounds **29** and **32** were specifically obtained from the liquid culture filtrate and exhibited antifungal activity [21, 22]. The structures of these compounds are shown in Fig. 1.

#### Linear alkynyl with C = 10

**Fig. 2:**
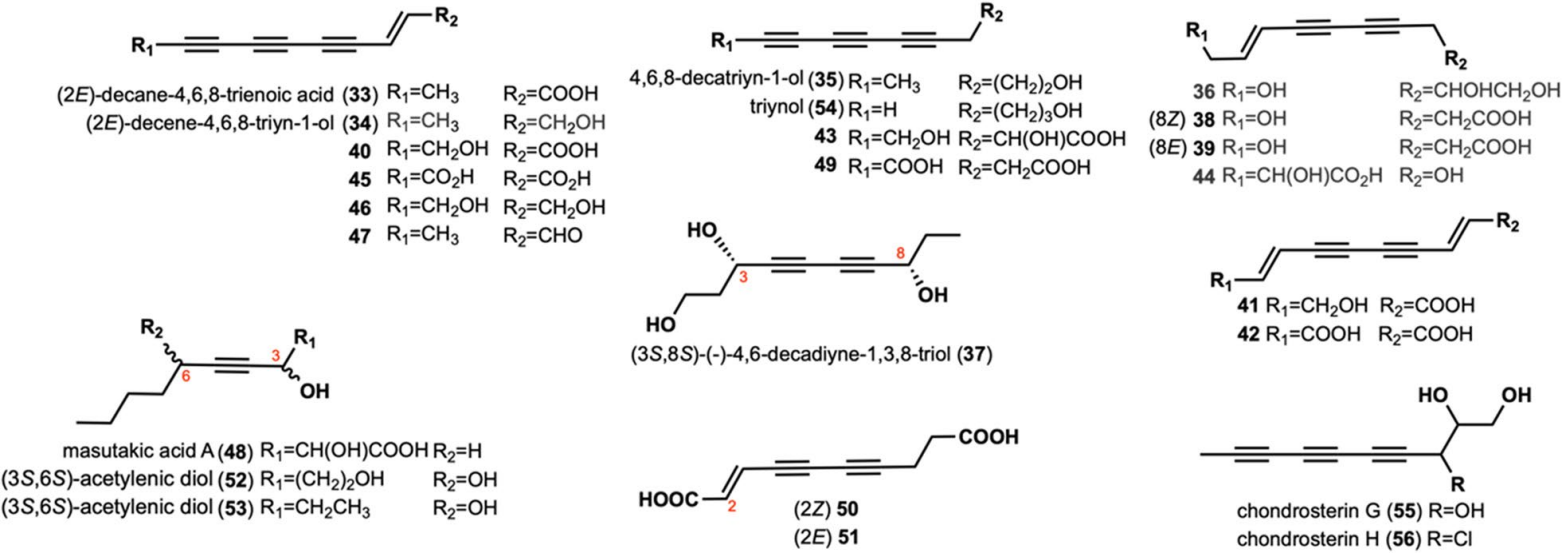
Chemical structures of linear alkynyl with C = 10

A total of 24 long-chain compounds consisting of 10 carbon atoms containing alkynyl groups were identified (Fig. 2). Two compounds, (2*E*)-decane-4,6,8-trienoic acid (**33**) and (2*E*)-decene-4,6,8-triyn-1-ol (**34**), were isolated from the fruit body of *Lyophyllum decastes*, a wild lotus leaf found in the Qilian Mountains [30]. These two compounds exhibit significant anti-oxidant activity against 2,2′-azobis (2-amidinopropane) dihydrochloride (ABAP) with EC_50_ values of 46.33 ± 3.48 μM/L, 65.6 ± 2.98 μM/L, respectively [30]. Notably, **34** was also identified from the metabolites of *Hypsizygus marmoreus* [31], *P. sinuosa* [16], *Lentinus lepideus*, *Leucopaxillus giganteus*, *Lyophyllurn dacastes* and *Peniophora resinosa* [32]*.* 4,6,8-decatriyn-1-ol (**35**) was isolated from *H. marmoreus* [31] and *P. scobinacea* [14]. Compound **36** was isolated from *H. marmoreus* [33]. (3*S*,8*S*)-(-)-4,6-decadiyne-l,3,8-triol (**37**) was isolated from *G. spectabilis* [34]*.* Compounds **38** and **39** were isolated from *F. pallida* [25]. Compounds **40**–**42** were found in *C. virgineus* [15]. Polyacetylenic compounds **43–46** were isolated from *P. sinuosa* [16]. Compound **47** was isolated from *L. lepideus*, *L. giganteus*, *L. dacastes*, and* P. resinosa* [32]. Masutakic acid A (**48**) was isolated from the fruiting bodies of *Laetiporus sulphureus* [35]. Compounds **49–51** were isolated from *S. lacrymans* [13]*.* Acetylenic diols, **52** and **53**, were isolated from the cultures of *C. catinus*, which exhibited anti-*B. subtilis* and anti-*B. cereus* activities at a concentration of 50 μg per disk [10]. Triynol (**54**) was isolated from the culture medium *P. scobinacea* [14]. Chondrosterin G (**55**) and chondrosterin H (**56**) were isolated from *Chondrostereum* species [36].

### Linear alkynyl containing amino or amide acids at the end

**Fig. 3:**
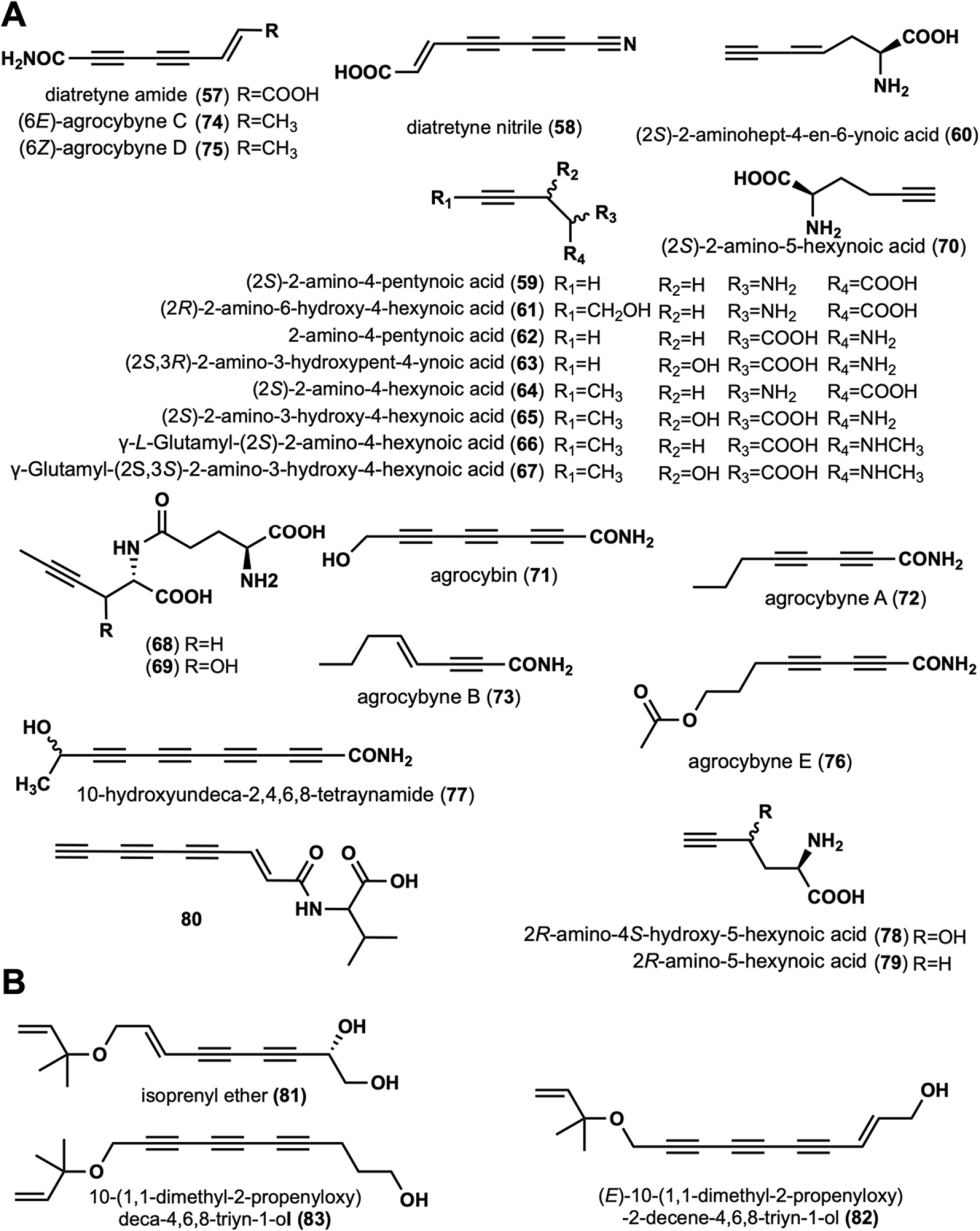
Chemical structures of linear alkynyl containing amino or amino acids at the end (**A**), and *O*-isopentenyl at the end (**B**)

These compounds are long-chain alkynyl structures with amino, carboxyl, and amide bonding modifications at the linear alkynyl ends. A total of 24 such compounds have been reported (Fig. 3A).

Diatretyne amide (**57**) and diatretyne nitrile (**58**) were isolated from cultures of *Clitocybe diatreta* and showed broad-spectrum antimicrobial activity [37]. Among them, **58** showed the most prominent inhibitory activity against *S. aureus* with a MIC value of 30 ng/mL [37]. In addition, mushrooms capable of producing **58** is *C. virgineus* [15]. (2*S*)-2-amino-4-pentynoic acid (**59**), (2*S*)-2-aminohept-4-en-6-ynoic acid (**60**) were isolated from the fruiting body of *Amanita pseudoporphyria* [38]. (2*R*)-2-amino-6-hydroxy-4-hexynoic acid (**61**) was isolated from *Amanita miculifera* [39]. Propargylglycine (2-amino-4-pentynoic acid, **62**), an acetylated amino acid, was identified in the poisonous *Amanita abrupta* [40], and it is hypothesized that **62** may inhibit fatty acid oxidation [40]. A toxic amino acid, (2*S*,3*R*)-2-amino-3-hydroxypent-4-ynoic acid (**63**) was isolated from *Sclerotium rolfsii,* and was lethal to New Hampshire chickens with LD_50_ of 150 mg/kg [41]. Six amino acid derivatives, (2*S*)-2-amino-4-hexynoic acid (**64**), (2S)-2-amino-3-hydroxy-4-hexynoic acid (**65**), γ-*L*-Glutamyl-(2*S*)-2-amino-4-hexynoic acid (**66**), γ-Glutamyl-(2*S*,3*S*)-2-amino-3-hydroxy-4-hexynoic acid (**67**), γ-glutamyl-L-2-aminohex-4-ynoic acid (**68**) and γ-L-glutamyl-L-erythro-2-amino-3-hydroxyhex-4-ynoic acid (**69**) were isolated from the fruiting body of *Tricholomopsis rutilans*. These amino acid derivatives have antiviral, anticholesterol, and anticancer activities [42–44]. (2*S*)-2-amino-5-hexynoic acid (**70**) was isolated from the fruiting body of *Cortinarius claricolor* var. *tenuipes* Hongo, which was characterized as a strong growth inhibitor against *B. subtilis* B-50 [45]. Agrocybin (**71**) was isolated from cultures of the fungi *Agrocybe perfecta* (Rick) Singer [46], *Agrocybe dura* [46], and *Cantharellus formosus* [47]. Intensive bioactivity investigations showed that **71** not only possessed trypanothione reductase inhibitory activity (IC_50_ 2 μM) but was also toxic to white mice (LD_50_ 6 mg/kg) [46]. It was also found to have significant activity against the human cancer cell lines UACC-62, MCF-7, and TK-10. In addition, **71** has phytotoxic, antibacterial, and antifungal activities [46, 48]. Agrocybynes A–E (**72**–**76**) were isolated from *Agrocybe praecox*, **72**–**75**, and were found to have significant growth inhibitory activity against lettuce [49]*.* 10-hydroxyundeca-2,4,6,8-tetraynamide (**77**) was isolated from *Mycena viridimarginata*, which demonstrates both antibacterial and antifungal activities [50]. Two unusual amino acids, 2*R*-amino-4*S*-hydroxy-5-hexynoic acid (**78**), and 2*R*-amino-5-hexynoic acid (**79**) were isolated from *Trogia venenata*, which showed low-dose toxic to mice [51]. Compound **80** was isolated and characterized from* P. sinuosa* [16]. The structures, names, biological activities, and sources of these compounds are shown in Fig. 3 and Table 1.

### Linear alkynyl containing O-isopentenyl at the end

This group contains compounds* O*-isopentenyl modifications, and has a backbone of at least two alkynyl groups. Isoprenyl ether (**81**) was isolated from *Fayodia bisphaerigera* [52]. (*E*)-10-(1,1-dimethyl-2-propenyloxy)-2-decene-4,6,8-triyn-1-ol (**82**) and 10-(1,1-dimethyl-2-propenyloxy)deca-4,6,8-triyn-1-ol (**83**) were isolated from *H. marmoreus* [31]. Their structures are displayed in Fig. 3B. No biological activity has been reported for any of these three compounds.

### Linear alkynyl with C > 10

These compounds are characterized by the presence of at least one alkynyl group and a linear chain of more than ten carbon atoms. Hydroxylation, carboxylation, and other modifications are present at their termini, and a total of 49 compounds have been identified.

#### Linear alkynyl with C11–C17

**Fig. 4:**
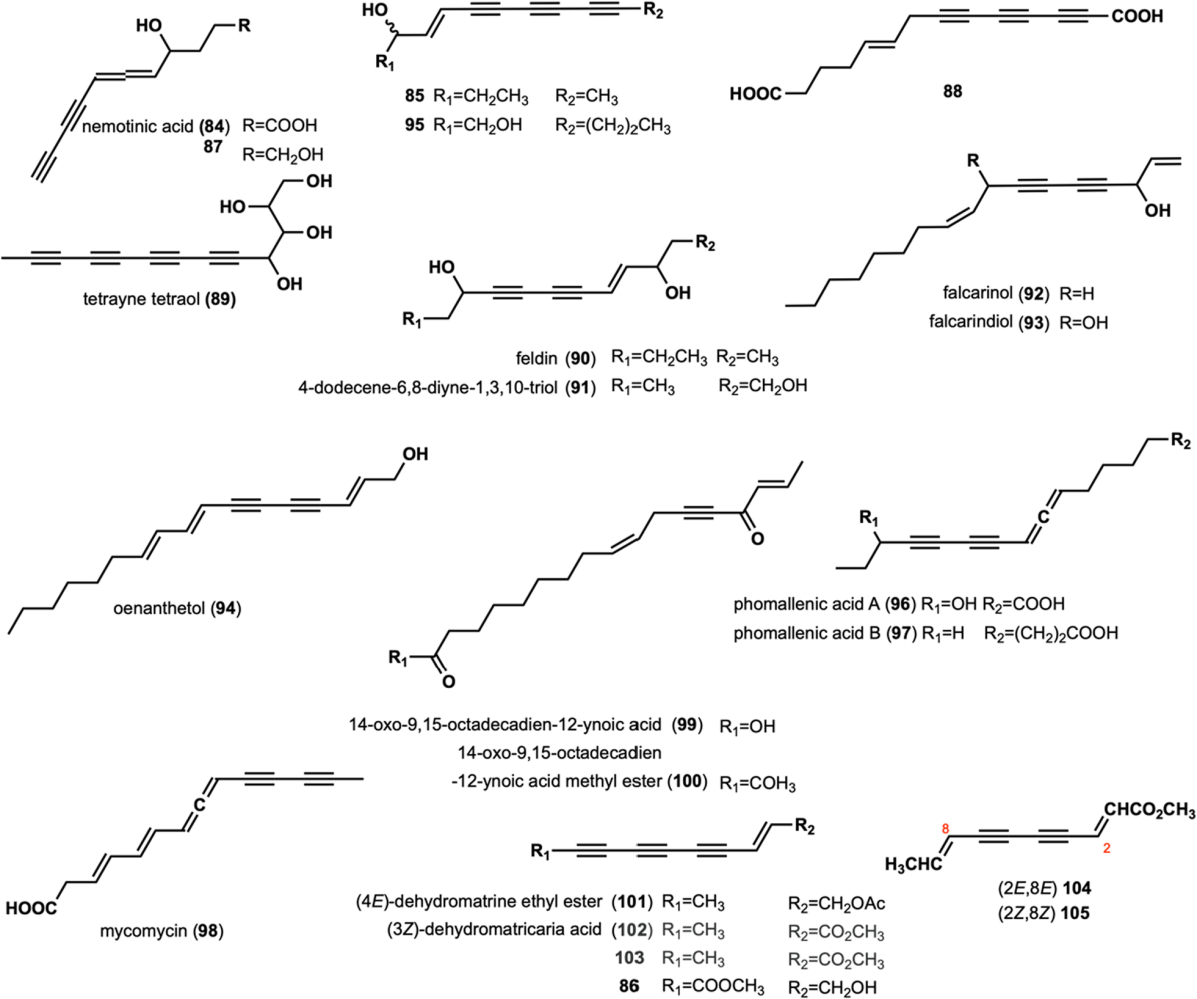
Chemical structures of linear alkynyl with C11–C17

These compounds consist of long chains of alkynyl groups of eleven to seventeen carbon atoms, containing up to four alkynyl groups. A total of 22 such compounds have been identified (Fig. 4).

Nemotinic acid (**84**) was isolated from Basidiomycete B-841, which exhibited antibacterial and antimycobacterial activities [28, 53–57]. Compounds **85–86** were separated from *L. lepideus*, *L. giganteus*, *L. dacastes*, and* P. resinosa* [32]. **87** was isolated from *Cortinellus berkeleyanus* [20]. Compound **88** was isolated and characterized from *P. sinuosa* [16]. Tetrayne tetraol (**89**) was isolated from the culture of *Fistulina hepatica* [25]. Five polyacetylenic compounds (**90–94**), namely Feldin, 4-dodecene-6,8-diyne-1,3,10-triol, falcarinol, falcarindiol, and oenanthetol, were also the metabolites of *F. hepatica* [58]. Triynene diol (**95**) was isolated from *F. pallida* [25]. Phomallenic acids A–B (**96**–**97)** were extracted from *Phoma* species [59], which showed type II fatty acid synthesis inhibitory activity [59]. Mycomycin (**98**) was isolated from *C. formosus* [47]. 14-oxo-9,15-octadecadien-12-ynoic acid (**99)** and 14-oxo-9,15-octadecadien-12-ynoic acid methyl ester (**100)** were isolated from *Cantharellus cibarius* [60]. Dehydromatrine ethyl ester (**101**) was isolated from the fruit body of *L. decastes* [30]. It exhibited significant anti-oxidant activity against 2,2′-azobis (2-amidinopropane) dihydrochloride (ABAP) with EC_50_ values of 43.4 ± 2.05 μM/L [30]. Dehydromatricaria acid (**102**) and compound (**103**) were isolated from the culture medium *P. scobinacea* [14]. Compounds **104** and **105** were isolated from *Polyporus* species [61].

#### Linear alkynyl with C = 18

**Fig. 5:**
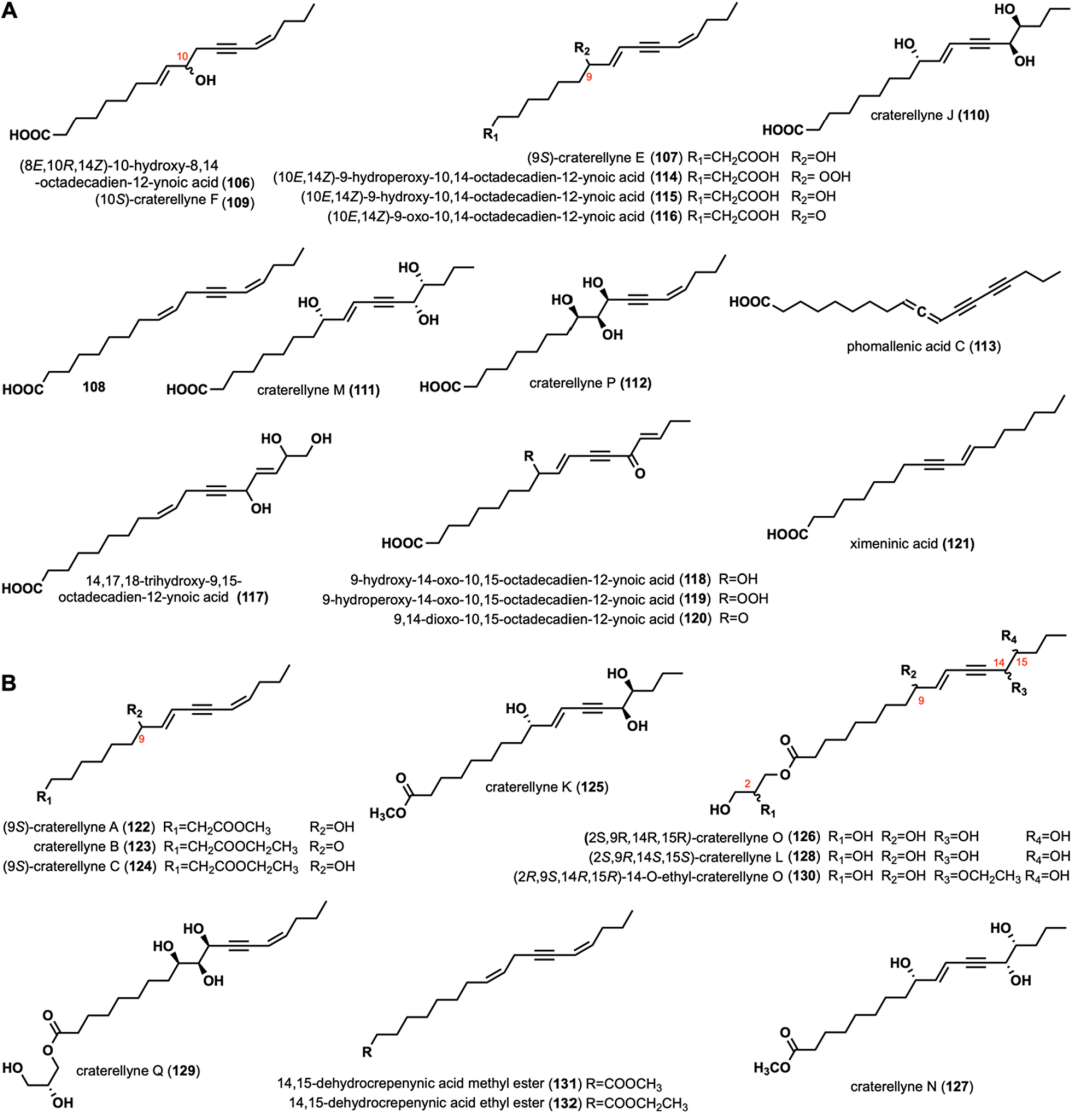
Chemical structures of linear alkynyl with C18 (**A**), and C > 18 (**B**)

These long-chain compounds containing alkynyl groups consist of eighteen carbon atoms, and sixteen compounds have been identified (Fig. 5A).

(8*E*,10*R*,14*Z*)-10-hydroxy-8,14-octadecadien-12-ynoic acid (**106**) was isolated from *Craterellus aureus* [62]. Craterellyne E (**107**), compound **108**, craterellyne F (**109**), craterellyne J (**110**), craterellyne M (**111**), and craterellyne P (**112**) were isolated from *Craterellus lutescens* [63, 64]. Phomallenic acid C (**113**) was extracted from *Phoma* species, which exhibited inhibitory against the wild-type *S. aureus* with the MIC of 3.9* μg*/mL[59]. (10*E*,14*Z*)-9-hydroperoxy-10,14-octadecadien-12-ynoic acid (**114**), (10*E*,14*Z*)-9-hydroxy-10,14-octadecadien-12-ynoic acid (**115**), (10*E*,14*Z*)-9-oxo-10,14-octadecadien-12-ynoic acid (**116**), 14,17,18-trihydroxy-9,15-octadecadien-12-ynoic acid (**117**), 9-hydroxy-14-oxo-10,15-octadecadien-12-ynoic acid (**118**), 9-hydroperoxy-14-oxo-10,15-octadecadien-12-ynoic acid (**119**), 9,14-dioxo-10,15-octadecadien-12-ynoic acid (**120**), ximeninic acid (**121**) were isolated from *C. cibarius*, [60, 65, 66]. Among them, **116** specifically activated PPAR-γ with an EC_50_ value of 1.88 μM [65].

#### Linear alkynyl with C > 18

These long-chain compounds containing alkynyl groups are composed of more than 18 carbon atoms, and eleven compounds have been identified (Fig. 5B).

Craterellynes A-C (**122**–**124**), craterellyne K (**125**), craterellyne O (**126**), craterellyne N (**127**), craterellyne L (**128**), craterellyne Q (**129**), and 14-*O*-ethyl-craterellyne O (**130**) were isolated from *C. lutescens* [63, 64]. In addition, **128** was also isolated from *C. aureus* [62]. 14,15-dehydrocrepenynic acid methyl ester (**131**) and 14,15-dehydrocrepenynic acid ethyl ester (**132**) were isolated from *C. cibarius* [60]. The structures, names, and sources of these compounds are shown in Fig. 5B and Table 1. Their activity investigations have not been reported.

### Linear alkynyl containing epoxy ring

**Fig. 6:**
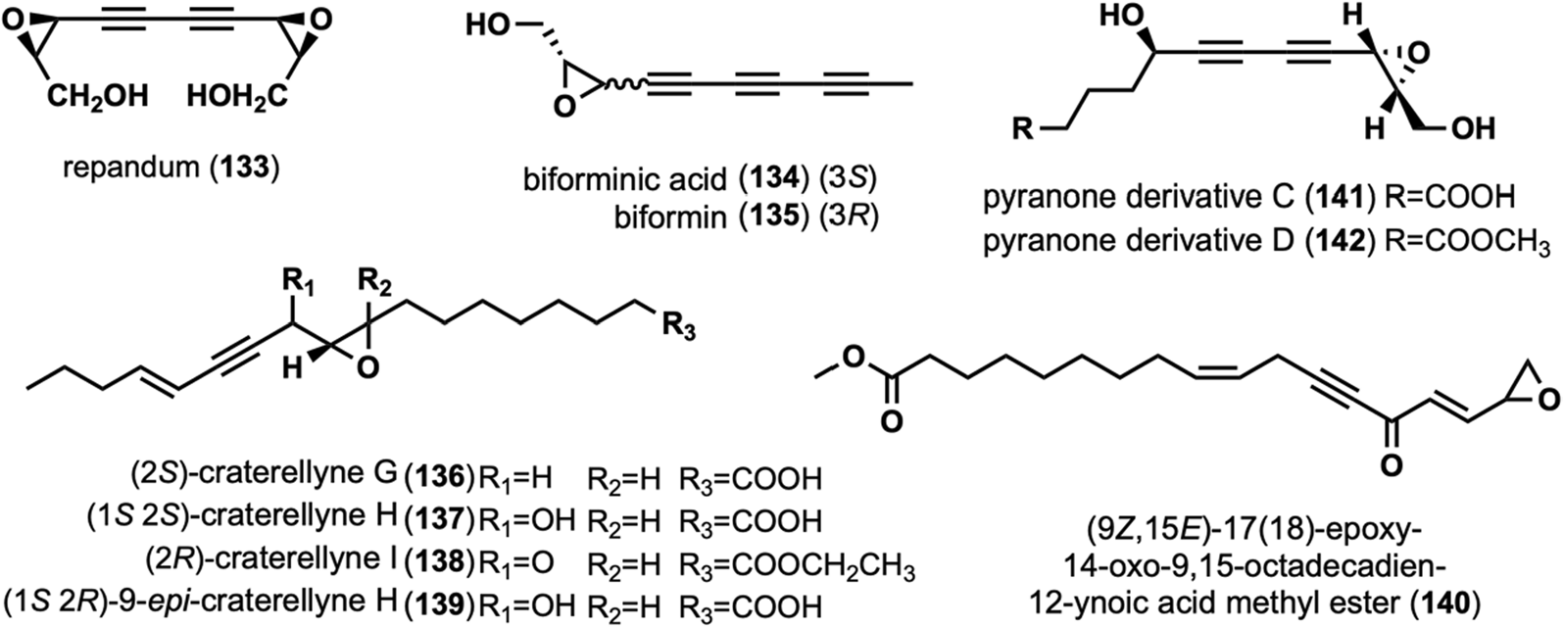
Chemical structures of linear alkynyl containing epoxy ring

These compounds are characterized by the presence of an epoxy ring structure in or at the end of a long chain containing an alkynyl group, and there are ten compounds in this group (Fig. 6).

The cytotoxic diepoxides, repandum (**133)** was isolated from *Hydnum repandum* [27, 67]. Compound **133** exhibited strong cytotoxic activity against various tumor cells [27], and **133** exhibited affinity for specific nucleic acids [27, 67]. Biforminic acid (**134)** and biformin (**135)** were obtained from *Polyporus biformis* [68], and has significant antibacterial activity against various bacteria [55, 68, 69]. Craterellynes G-I (**136–138**), along with 9-epi-craterellyne H (**139**), were isolated from *C. lutescens* [63]. Among them, **138** was cytotoxic to human cancer cells, inhibits nitric oxide (NO) production, and has weak antibacterial activity [63]. (9*Z*,15*E*)-17(18)-epoxy-14-oxo-9,15-octadecadien-12-ynoic acid methyl ester (**140**) was isolated from *C. cibarius *[60]. Pyranone derivatives C–D (**141**–**142)** were isolated during the study of the chemical composition of* J. nitida*, and activity evaluation revealed that they exhibited toxic cytotoxicity (IC_50_ greater than 40 μM) against five human cancer cell lines, including Human myeloid leukemia HL-60 [24].

### Linear alkynyl containing lactone ring

**Fig. 7:**
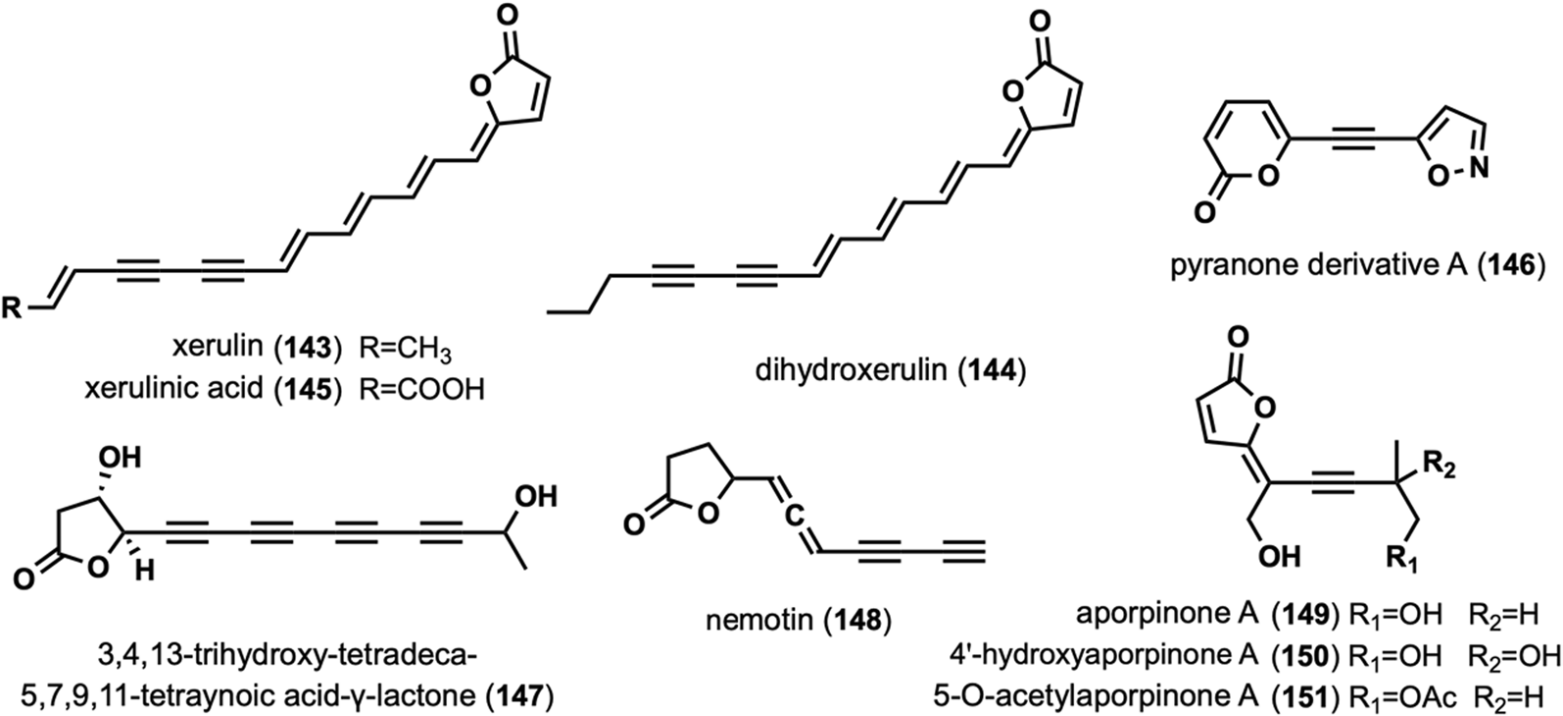
Chemical structures of linear alkynyl containing lactone ring

These alkynyl-containing compounds are characterized by the presence of a lactone ring at the end of the linear chain, and a total of nine such compounds have been identified (Fig. 7). Studies of the chemical composition of *Xerula melanotricha* [70], *F. hepatica* [58] led to the isolation of three highly unsaturated, intensely yellow γ-alkylidene butenolides (**143**–**145**), (xerulin, dihydroxerulin, and xerulinic acid), which inhibited the suppression of cholesterol biosynthesis in human HeLa S3 cells by targeting HMG-SCoA synthase [70]. Pyranone derivative A (**146**) was isolated from *J. nitida* [24], and toxicity evaluation revealed IC_50_ values ranging from 4.13 to 11.65 μM for human myelogenous leukemia HL-60, hepatocellular carcinoma SMMC-7721, cancer A-549, breast cancer MCF-7 and colon cancer SW480 [24]. 3,4,13-trihydroxy-tetradeca-5,7,9,11-tetraynoic acid-γ-lactone (**147**) was isolated from *M. viridimarginata* [50]. Nemotin (**148**) was isolated from *C. formosus* [47]. Aporpinone A (**149**) was isolated from *Aporpium caryae* [71] and *Hexagonia speciosa* [72], and showed inhibitory activity against three cell lines, including SMMC-7721, A-549 and MCF-7 [72]. 4ʹ-hydroxyaporpinone A (**150**) was isolated from the basidiomycetes woody fungus *A. caryae* [71]. 50-o-acetylaporpinone A (**151**) was isolated from *H. speciosa* [72].

### Linear alkynyl containing six-membered ring structure

**Fig. 8:**
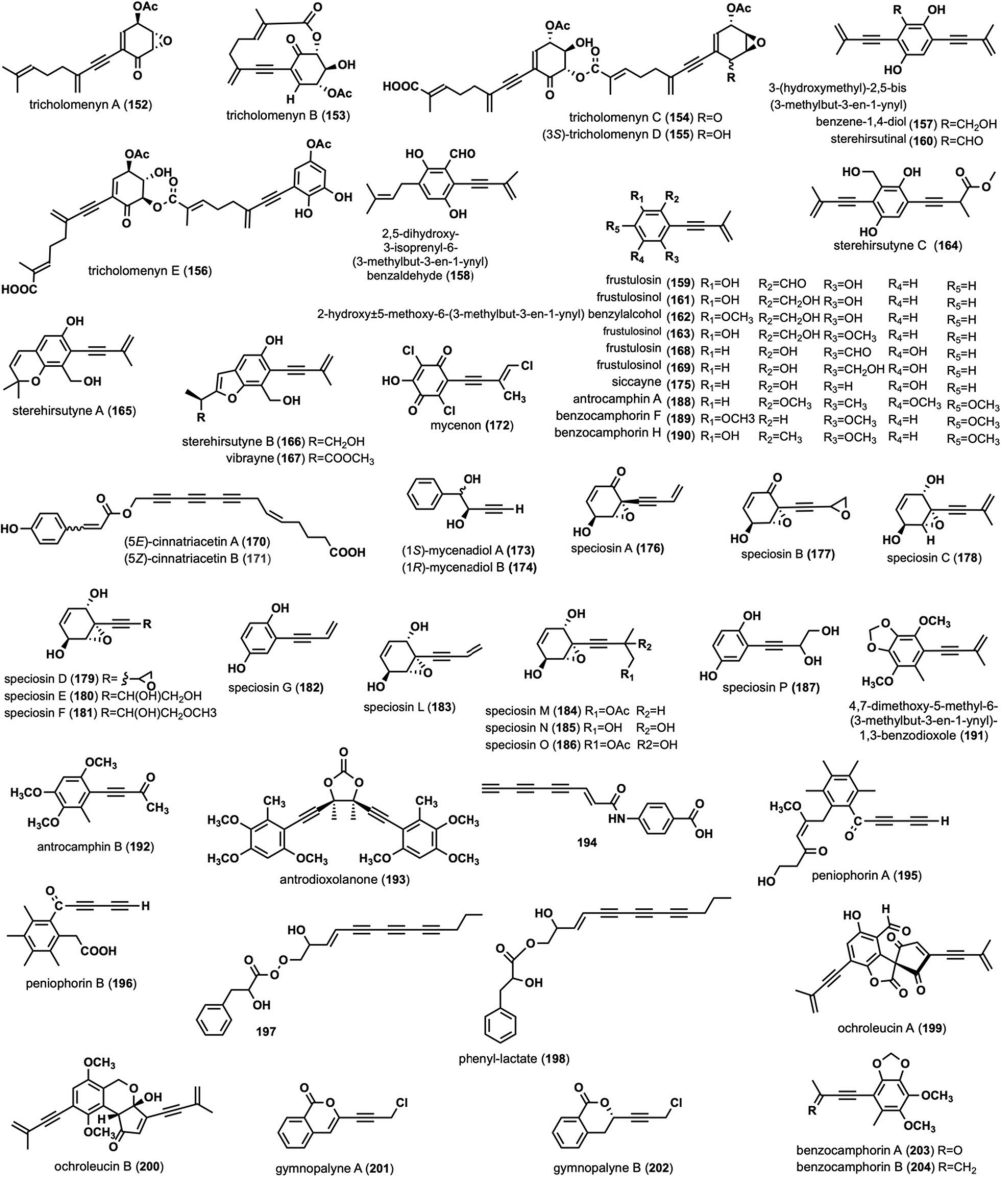
Chemical structures of linear acetylene containing six-membered ring structure

These compounds are characterized by long alkynyl chains containing six-membered ring. Linear alkynyls in these compounds are modified with various functional groups, such as carboxylation, carbonylation, hydroxylation, chlorination, aldehyde, acetylation, etc. The majority of these compounds contain one to three alkynyl groups. A total of 53 compounds of this type have been identified (Fig. 8).

Tricholomenyns A–E (**152**–**156**) were isolated from *Tricholoma acerbum* [73, 74]. Tricholomenyns effectively inhibits mitosis in T lymphocyte cultures and is a potent anticancer agent [73]. Compounds **152** and **153** exhibited a significant inhibitory effect on mitosis in T lymphocyte cultures [73]. Compound **154** was found not only in *T. acerbum* but also in other species like *T. ustaloides* Romagn [74], *T. vaccinum* Kummer [74], *T. albobrunneum* Kummer [74], and *T. imbricatum* Kummer [74], suggesting it may serve as a useful chemotaxonomic marker for many species within Tricholoma [74]. 3-(hydroxymethyl)-2,5-bis(3-methylbut-3-en-1-ynyl)benzene-1,4-diol (**157**), 2,5-dihydroxy-3-isoprenyl-6-(3-methylbut-3-en-1-ynyl)benzaldehyde (**158**), frustulosin (**159**), sterehirsutinal (**160**), frustulosinol (**161**), 2-hydroxy-5-methoxy-6-(3-methylbut-3-en-1-ynyl) benzylalcohol (**162**), frustulosinol (**163**), sterehirsutyne C (**164**), sterehirsutynes A (**165**)–B (**166**) and vibrayne (**167**) were isolated and characterized from the cultures of the fungus *Stereum hirsutum* [75, 76]. Compound **159** demonstrates potent phytotoxicity by causing wilting of stems and leaves and inhibiting callus growth, with 100 μM concentration inhibiting 50% and 500 μM inhibiting 100% of callus growth [75]. Compounds **164** and **165** exhibited moderate inhibitory activity against porcine pancreatic lipase (PPL), with IC_50_ values of 21.8 ± 2.15 μM and 23.2 ± 1.04 μM, respectively [76]. Frustulosin (**168)** and frustulosinol** (169)** were obtained from *Stereum frustulosum* [77]. Compound **168** demonstrated activity against *S. aureus*, *Bacillus mycoides*, and *B. subtilis* at a concentration of 16 ppm, while also displaying moderate activity against *Vibrio cholera* and *V. cholera* phage. Compound **169** exhibited activity at a concentration of 16 ppm against *S. aureus* and at a range of 64–256 ppm against *Mycobacterium smegmatis* [77]. Cinnatriacetins A (**170**) and B (**171**) were extracted from *F. hepatica*, and compounds **170**–**171** exhibited antimicrobial activities [78]. 20 μg of compound **170** on 8 mm paper disks inhibited *S. aureus* IFO 12732, *B. subtilis* ATCC 6633, *B. cereus* 1AM 1110, and *Bacillus coagulans* IFO 1 with zone diameters of 13.4 mm, 14.0 mm, 11.6 mm, and 14.2 mm, respectively [78]. Compound **171**, under the same conditions, inhibited the same bacteria with zone diameters of 12.4 mm, 13.2 mm, 11.8 mm, and 13.6 mm, respectively [78]. Mycenon (**172**) was isolated from *Mycena species*, showing novel inhibitory activity against isocitrate lyase (EC 4.1.3.1) [79]. Compound **172** is also active against both bacteria and fungi [79]. Mycenadiols A–B (**173**–**174**) were isolated from the culture of *Mycena pruinosoviscida* BCC 22723 [80]. Siccayne (**175**) was obtained from ascomyces [81]. Compound **175** disrupts the uptake of nucleoside precursors by eukaryotic cells and inhibits the incorporation of nucleotides into DNA and RNA in vitro [81]. Speciosins A-G (**176**–**182**) and speciosins L-P (**183**–**187**) were isolated from *H. speciosa* [72, 82]. Compound **177** exhibited significant inhibitory activity against all five cell lines, with IC_50_ values of 0.23 μM (human myeloid leukemia HL-60), 0.70 μM(hepatocellular carcinoma SMMC-7721), 3.30 μM (lung cancer A-549), 2.85 μM (breast cancer MCF-7) and 2.95 μM (colon cancer SW480), respectively [72]. Antrocamphin A (**188**), benzocamphorin F (**189**), benzocamphorin H (**190**), 4,7-dimethoxy-5-methyl-6-(3-methylbut-3-en-1-ynyl)-1,3-benzodioxole (**191**), antrocamphin B (**192**) were isolated from *Antrodia cinnamomea* [83–86]. Compound **188** displayed strong anti-inflammatory activity against LPS-challenged macrophages [87]. At a concentration of 20 μg/mL, it normalized NO and PGE2 levels, dose-dependently suppressed both iNOS and COX-2 protein and mRNA expression (at concentrations of 1–20 μg/mL), and hindered the translocation of NF-κB to the nucleus [87]. Additionally, compound **188** exhibited an effective inhibitory effect on fMLP-induced Superoxide production, IC_50_ < 10 μM [83]. Furthermore, compound **188** was also isolated from *Taiwanofungus camphoratus* and displayed moderate cytotoxicity with an ED_50_ = 3.4 μg/mL against MCF-7 and Hep2 cell lines [88]. Benzocamphorin F (**189**) exhibited potent nitric oxide synthase (NOS) inhibitory activity and mild NADPH oxidase (NOX) inhibitory activity in mouse Microglia cells (BV2 cells) [85]. Benzocamphorin H (**190**) exhibited moderate anti-inflammatory activity by inhibiting LPS-stimulated RAW 264.7 macrophages, with an IC_50_ of 15.09 ± 1.21 M in nitrite production [84]. **191** displayed anti-inflammatory activity by inhibiting superoxide anion production and human neutrophils to N-formylmethionyl-leucyl-phenylalanine (FMLP)/cytochalasin B [86]. Antrodioxolanone (**193**) was extracted from *Antrodia camphorata* [83] and *T. camphoratus* [88]. Compound **194** was obtained from *Baeospora myosura* [89] and *C. formosu*s [47], and it exhibited strong inhibition against gram-positive bacteria, particularly *S. aureus* (MIC = 0.001 μg/mL) [89]. Two types of polyacetyl antibiotics-Peniophorins A (**195**) and B (**196**) were isolated from *Peniophora affinis* [90]. Compounds **195** and **196** exhibit antimicrobial activities [90]. Compounds **195** and **196** have

### Other types of compounds with alkynyl structure

#### Linear alkynyl containing indole ring structure

**Fig. 9:**
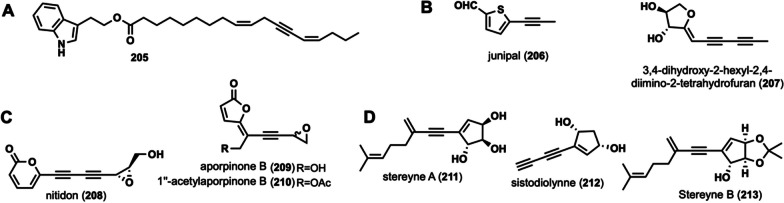
Chemical structures of other linear alkynyl compound. Chemical structures of linear alkynyl containing indole ring (**A**), Chemical structures of linear alkynyl containing S or O (**B**), Chemical structures of linear alkynyl containing both lactone ring and epoxy ring (**C**), and Chemical structures of linear alkynyl containing a five-ring structure (**D**)

Such compounds are long alkyne-based chains containing an indole ring with an alkyne group. Compound **205** (Fig. 9A) is currently the only natural compound with these characteristics from a mushroom isolated from *Craterellus cornucopioides* [92]. Although **205** was identified as early as 1989, its activity is not yet known.

#### Linear alkynyl containing sulfur, oxygen structure

These compounds are alkynyl chains containing  a thiophene ring (S) or a furan ring (O). Junipal (**206**) was isolated from the fungus *D. juniperina* [93]. 3,4-Dihydroxy-2-hexyl-2,4-diimino-2-tetrahydrofuran (**207**) was isolated from* L. decastes* and possessed strong anti-oxidant activity with an EC_50_ value of (24.73 ± 6.12) μM/L [30]. Their structures are shown in Fig. 9B.

#### Linear alkynyl containing both lactone ring and epoxy ring

The class of compounds refers to alkynyl long chains containing lactone ring and epoxy ring, with a carbonyl group, hydroxylation, and other modifications at the end. There are three compounds, with most of them containing one or two alkynyl groups.

Nitidon (**208**), obtained from *J. nitida* [24], induces both morphological and physiological differentiation in HL-60 and U-937 tumor cell lines [94]. Additionally, it displays antibacterial and antifungal properties and exerts cytotoxic effects against HL-60 and U-937 cells at 250 ng/mL, as well as against L1210, HeLa, S3, and BHK-21 cells at 500 ng/mL [94]. Aporpinone B (**209**) and 1''-acetylaporpinone B (**210**) were isolated from *A. caryae* [71]. Compounds **209** and **210** displayed modest antibacterial activity when assessed using the agar diffusion method against *B. subtilis*, *S. aureus*, and *E. coli* [71]. Their structures are shown in Fig. 9C.

#### Linear alkynyl containing a five-ring structure

This class of compounds consists of long chains of alkynyl groups with a pentacyclic ring structure, featuring hydroxylation modifications at the end. There are three subtypes of these compounds. Stereyne A (**211**) was isolated from *S. hirsutum* [95]. Sistodiolynne (**212**) was isolated from *Sistotrema raduloides* [96]. Stereyne B (**213**) was isolated from *S. hirsutum* [95]. Their structures are shown in Fig. 9D. Their activity investigations have not been reported.

## Discussion and perspective

Mushrooms have garnered considerable attention as a valuable source of bioactive compounds used in the development of dietary supplements and pharmaceuticals. This article reviews recent advances in discovering alkynyl compounds in mushrooms over the last 70 years. We have summarized the chemical structures and biological activities of 213 alkynyl-containing compounds isolated from fungi. We have categorized these alkynyl compounds into fourteen groups based on the number of carbon atoms and characteristic structures. This categorization provides a convenient reference for future discovery of compounds with similar structures or different modifications. In addition, we have summarized the structural diversity and biological activities of these compounds, providing robust options for potential future market applications based on academic research and theoretical insights.

With the advancement of gene sequencing technology, an increasing number of mushroom genomes [97–100] have been sequenced and made publicly available. A survey has revealed that over a dozen mushroom genomes that produce alkynyl compounds have been published. Among these genomes, those that have already been reported include *A. cinnamomea* [101], *L. edodes* [102], *H. marmoreus* [103], *G. spectabilis* [104], *S. hirsutum* [105], *Lentinus tigrinus* [106], *S. lacrymans* [107], *L. sulphureus* [108], *Trametes pubescens* [109], *F. hepatica* [110], *R. ochroleuca* [111]. The publication of these genomes provides essential sequence information for the biosynthesis of related alkynyl compounds.

Identified alkynyl compounds from mushrooms exhibit significant biological activities, including antibacterial, antifungal, insecticidal, cytotoxic, phototoxic, anticancer, and anti-oxidant activities. Among these, antibacterial activity is the most widespread and stands out as one of the prominent types among natural products. However, the activity of most alkynyl compounds remains unknown, and a comprehensive investigation of these compounds will lay the foundation for exploring their potential medicinal resources. As research on alkynyl compounds in mushrooms continues to expand and deepen, more bioactive metabolites can be isolated. The elucidation of the structures of these compounds is crucial for a deeper understanding and exploitation of the potential of this class of compounds.

## Data Availability

Not applicable.
